# Real world insights on the initiation and treatment duration of oral antiplatelets in acute coronary syndromes: a retrospective cohort study

**DOI:** 10.1093/ehjcvp/pvw043

**Published:** 2017-01-24

**Authors:** Marc J. Claeys, Christophe Beauloye, Suzanne Pourbaix, Peter R. Sinnaeve, on behalf of Rewinder Study Group

**Affiliations:** 1 Department of Cardiology, Antwerp University Hospital, Wilrijkstraat 10, 2650 Edegem, Belgium; 2 Institut de Recherche Expérimentale et Clinique, Pôle de Recherche Cardiovasculaire, Université Catholique de Louvain, Prom. de l’Alma, 1200 Woluwe St-Lambert, Brussels, Belgium; 3 Service de Cardiologie, Centre Hospitalier Régional de la Citadelle, Boulevard du Douzième de Ligne 1, 4000, Liège, Belgium; 4 Department of Cardiovascular Sciences, University Hospitals Leuven, O&N I Herestraat 49, 3000, Leuven, Belgium

**Keywords:** Oral antiplatelet therapy, Acute coronary syndrome, Treatment persistence, Prasugrel, Ticagrelor, Clopidogrel

## Abstract

**Aims:**

This study is a real world, observational study evaluating the treatment persistence of oral antiplatelet (OAP) therapy during a one-year follow-up in patients after an acute coronary syndrome (ACS).

**Methods and results:**

Data on diagnosis, comorbidities, follow-up, OAP treatment, reasons, and decision maker for treatment discontinuation in patients who were discharged from a hospital in Belgium after an ACS between 1 July 2012 and 1 June 2013 were collected by cardiologists from 18 centres, up to 360 days from discharge. Out of the 671 patients surveyed, 295 patients were included in the persistence analysis. The remainder was excluded from the analysis due to the lack of precise information on OAP stopping date. The proportion of patients still using OAPs after 90, 180, 270, and 360 days was 92, 89, 83, and 73%, respectively. OAP persistence was higher for patients treated with prasugrel or ticagrelor. At 360 days, 79% of patients with a ST-segment elevation myocardial infarction (STEMI) and 66% of patients with a non-STEMI were still adhering to the prescribed course of treatment. Among the 79 patients with early treatment discontinuation, the mean treatment duration was 197.0 ± 125.18 days. The main decision taker in premature treatment cessation was the cardiologist (31% of cases), while the most frequently cited reasons included surgery (25%) and perceived high bleeding risk (19%).

**Conclusion:**

Treatment persistence with OAPs after ACS in Belgium is high throughout the recommended period. Discontinuation was observed more often in patients treated with clopidogrel and was mainly initiated by the cardiologist.

## Introduction

Acute coronary syndromes (ACSs) represent a life-threatening range of clinical conditions that are almost always associated with the rupture of an atherosclerotic plaque and partial or complete thrombosis of the infarct-related artery. Platelet aggregation, induced by plaque rupture, is an important contributor to the generation of atherothrombotic events.^1^

Dual antiplatelet therapy (DAPT) consisting of aspirin and a P2Y_12_ receptor inhibitor is the recommended treatment following an ACS. Guidelines include maintaining a long-term DAPT course for one year after an ACS event.^2–4^ Early discontinuation of DAPT has been associated with adverse outcomes. A retrospective observational cohort study from the UK assessing clopidogrel therapy persistence in a population of 4650 patients discharged after acute myocardial infarction (MI) found that premature discontinuation of clopidogrel during the first 12 months of treatment was associated with a significant increase in the risk of death or recurrent infarction.^5^

In Belgium, a full year of DAPT (either clopidogrel, prasugrel, or ticagrelor) is universally reimbursed following an ACS. To date, however, there is little information on oral antiplatelets (OAPs) treatment persistence after an ACS among Belgian patients. The current study (REal World insights on the INitiation and treatment Duration of ticagrEloR and other OAPs in patients with ACS in Belgium [REWINDER]) aimed at retrospectively collecting data on the treatment persistence with OAPs after an ACS, and the reasons and deciders of OAP treatment switch, discontinuation, or re-initiation.

## Methods

### Study design

The study was a real world, multicentre, noninterventional, retrospective study carried out in 18 medical centres in Belgium between 4 September 2014 and 30 January 2015, among patients treated with OAPs after a hospitalization for an ACS.

All data were collected from medical records, for a period of 1 year after discharge from the hospital following the event. If the follow-up period was not fully covered by the available files, the cardiologists were required to contact the general practitioner (GP) or to consult hospital records in order to complete the case report files.

The OAPs targeted by the study were clopidogrel (*Plavix*™), prasugrel (*Efient™*), or ticagrelor (*Brilique*™). Currently, clopidogrel, and ticagrelor are reimbursed by the social security in their registered indication in Belgium. Clopidogrel is indicated in adults suffering with MI, ischaemic stroke, established peripheral arterial disease, ACS, ST-segment elevation myocardial infarction (STEMI), or non-STEMI (in combination with acetylsalicylic acid). Ticagrelor co-administered with acetylsalicylic acid is indicated for the prevention of atherothrombotic events in adults with ACS or a history of MI and a high risk of developing an atherothrombotic event. Prasugrel is only reimbursed in STEMI patients undergoing percutaneous coronary intervention (PCI), diabetic NSTEMI patients, and patients with a stent thrombosis. All drugs are explicitly reimbursed for a period of 1 year after an ACS event.

Prior to data collection, all patients were informed of the study through an information letter. The study was designed and conducted according to the Declaration of Helsinki, Good Clinical Practice and local regulations and is registered at www.clinicaltrials.gov (NCT02190123).

The study protocol was approved by local ethics committees.

### Study population

The patients enrolled in the study were men and women over 18 years, discharged alive from the hospital following an ACS event diagnosed with STEMI, NSTEMI, or unstable angina (UA) that had occurred between 1 July 2012 and 1 June 2013. The ACS event was required to have been either UA or MI of type 1 (spontaneous MI related to ischaemia due to a primary coronary event such as plaque erosion and/or rupture, fissuring, or dissection) in order to be included in the study. Eligible patients were receiving one of the 3 OAPs at discharge: clopidogrel, ticagrelor, or prasugrel. Only patients with documented use of OAPs and/or exact discontinuation dates were analysed, regardless of the source of the information (medical records from cardiologist or GP).

The patients were considered ineligible if they had participated in any interventional clinical study during the REWINDER observation period, if the ACS event occurred during a stay in the hospital or if the ACS event was precipitated by or arose as a complication of surgery, trauma, gastrointestinal bleeding or PCI.

### Study objectives

The primary objective of the study was to evaluate the actual treatment persistence with OAPs up to 1 year after an ACS in the clinical practice of Belgium. Secondary objectives were: (i) to describe the most frequent reasons for OAP treatment switch, discontinuation, or re-initiation; (ii) to identify the persons (patient, interventional, or non-interventional cardiologist, GP, other) who asked for or decided on the OAP treatment switch, discontinuation, or re-initiation; and (iii) to assess distinct patient profiles associated with premature treatment discontinuation. Treatment switch was defined as a change from the index OAP to another OAP targeted by the study.

Owing to the non-interventional character of this study, no pro-active safety data collection took place.

### Statistical analysis

Demographics and baseline characteristics analyses were performed on the study population containing all evaluable patients, i.e. patients for whom follow-up data on OAP treatment were available at least 11 months after discharge either through hospital or GP records. Only patients for whom the status was clear at 360 days and for whom the treatment stop date was known were included in the treatment persistence analysis.

Analyses consisting of simple frequencies and descriptive statistics of all variables were carried out. Cross-tabulations of variables were performed when relevant. Patients’ demographics and baseline characteristics were tabulated overall, by gender and stratified by 2 age categories: <65 years and ≥65 years.

The relationship between overall OAP treatment persistence rate and multiple factors was analysed using multivariate logistic regression models, with age, gender, hospital type, ACS diagnosis and management, cardiovascular (CV) history, CV risk factor, OAP treatment, and concomitant medication at discharge included as independent variables. Adjusted *P*-values were computed for each analysis.

Statistical significance was defined as *P* < 0.05 and all tests were 2-sided. Univariate analyses were carried out using the Statistical Package for the Social Sciences (SPSS version 13.0, IBM). Multivariate analyses were performed using the Statistical Analysis Software (SAS).

The non-randomized, retrospective design of this study cannot support any formal cross-therapy comparisons with respect to the endpoint variables. Hence, all analyses are considered exploratory and the *P*-values will be interpreted descriptively.

## Results

### Demographics

**Figure 1:**
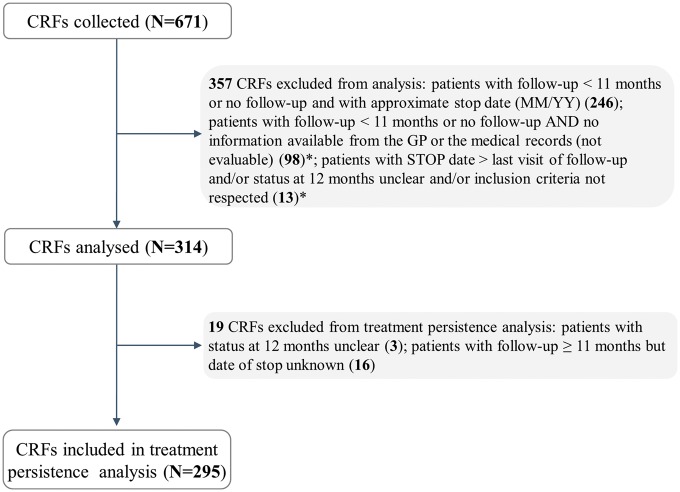
Patients’ flow chart. CRF, case report form; *n*, number of patients. Asterisk indicates no information available at 12 months, therefore the CRFs were excluded from the analysis.

Eighteen PCI and non-PCI hospitals from Belgium were included in the study: 39% of the participating sites were academic PCI centres, 33% were non-academic PCI centres, and 28% were non-academic non-PCI centres. A total of 671 case report files were screened and 314 were finally included in this analysis (total study population). The reasons for exclusion are presented in *Figure *1. An additional 19 patients were excluded from the treatment persistence analysis: 3 patients with an exact date for the initial OAP treatment stop, but with unclear status at 12 months, after OAP re-initiation and 16 patients with a follow-up of more than 11 months, but without an exact date of treatment stop (*Figure *1).

Demographic characteristics for the total study population (*n* = 314) are given in *Table *1. Approximately one-half of the patients belonged to the <65 years age group. The majority of patients were men, with an average age of 63.4 ± 11.7 years (mean age ± standard deviation) at the time the ACS occurred, while female patients were older (67.5 ± 13.3). The average age among all patients at the onset of the ACS event was 64.4 ± 12.2 years. The cohort for treatment persistence was similar in terms of demographics, cardiovascular history, final diagnosis, and established course of treatment to an analysed study population including 560 case report forms with OAP treatment status recorded according to a less stringent certainty criterion (i.e. lacking exact information at 12 months; *Figure *1).

**Table 1:** Baseline characteristics for the study population (*n* =  314)

	Count	%
*Age category*		
<65 years	164	52
≥65 years	150	48
*Gender*		
Male	240	76
Female	74	24
*Type of hospital*		
Academic PCI	127	40
Non-academic PCI	91	29
Non-academic non-PCI	96	31
*ACS type*		
Unstable angina	31	10
STEMI	160	51
NSTEMI	123	39
*ACS management*		
PCI	286	91
CABG	6	2
PCI+CABG	4	1
Angiography without coronary intervention	7	2
Non-invasively managed (medically managed)	11	4
*OAP*		
Clopidogrel	99	32
Prasugrel	60	19
Ticagrelor	155	49

*n*, number of patients in the study population; PCI, percutaneous coronary intervention; ACS, acute coronary syndrome; STEMI, ST-segment elevation myocardial infarction; NSTEMI, non-ST-segment elevation myocardial infarction; CABG, coronary artery by-pass grafting; OAP, oral antiplatelet.

### Diagnosis, treatment, and CV history and risk factors

Half of the total patients (160/314; 51%) were diagnosed with STEMI, while only a minority of patients (31/314; 10%) were diagnosed with UA (*Table *1). The mean age of patients at the time of the ACS event was 63.3 ± 12.4 years for patients diagnosed with STEMI and 66.3 ± 12.1 years for patients diagnosed with NSTEMI.

The prescribed OAP at hospital discharge was clopidogrel for 32% of the patients, prasugrel for 19% and ticagrelor for 49% of the study population. Patients on concomitant oral anticoagulation treatment at discharge (24/314; 8%) received more frequently clopidogrel (11/24; 46%) and ticagrelor (10/24; 42%) than prasugrel (3/24; 8%).

The CV history and risk factors among the patients are shown in *Table *2. More than half of the patients (59%) reported no past CV diseases, while prior coronary artery diseases were documented in 193 instances. Only 8% of the study population had a prior history of peripheral vascular disease. The most prevalent CV risk factor was dyslipidaemia or treatment with cholesterol lowering drugs (225/314; 72%).

**Table 2:** CV history and risk factors for the study population (*n* = 314)

	Count	%^a^
*CV history*		
None	184	59
Prior CAD^b^	193	61
Cardiac heart failure	12	4
Atrial fibrillation	19	6
Stroke/transient ischaemic attack	16	5
Peripheral vascular disease	24	8
Major bleeding events	2	1
Other CV history	8	3
*CV risk factors*		
None	19	6
Arterial hypertension or antihypertensive drugs	198	63
Dyslipidemia or cholesterol lowering drugs	225	72
Diabetes or hypoglycaemic therapy	72	23
Active smoker	126	40
Familial history of coronary artery disease	95	30
Obesity (BMI >30)	55	18
Chronic kidney disease (GFR <60 mg/dl/min)	29	9
Other CV risk factors	2	1

*n*, number of patients; CV, cardiovascular; CAD, coronary artery disease; BMI, body mass index; GFR, glomerular filtration rate.

a Total percentages are higher than 100 due to multiple responses possible per patient.

b Prior CAD includes prior myocardial infarction, prior percutaneous coronary intervention, prior coronary artery bypass graft, coronary artery disease and stenosis >50%.

### Follow-up visits

On average, the patients were visiting their cardiologist almost 4 times (3.7 ± 1.6) during the year following discharge. Half of the patients 163/314 (52%) had between 1 and 3 visits, 129/314 (41%) had between 4 and 6 visits and only 2/314 (1%) had up to 10–12 visits to the hospital or cardiologist during the follow-up period. The mean time interval between discharge and the last visit to hospital was 11.0 ± 2.7 months after discharge and the interval between hospital discharge and visit 1 was 1.1 ± 1.6 days (calculated for *n* = 308 patients).

### Treatment persistence

**Figure 2:**
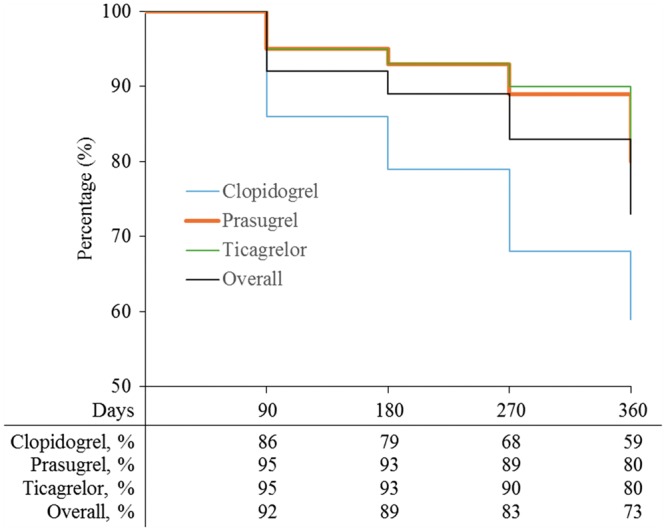
Persistence of medication during the treatment course for the analysed population (*n* = 295), by OAP type and overall. *n*, number of patients; OAP, oral antiplatelet.

The treatment persistence analysis included 295 patients out of the 314 included in the study population (*Figure *1). The measured treatment persistence with OAP after an ACS in the clinical practice of Belgium at 360 days from hospital discharge was 216/295 (73%) (*Figure *2). For the 79 patients who stopped treatment before 360 days, the mean treatment duration was 197.0 ± 125.18 days (median 216 days).

At 90, 180, and 270 days, respectively, the proportion of patients who were still using OAPs was 92, 89, and 83%. Treatment persistence for clopidogrel was lower compared with prasugrel or ticagrelor (*Figure *2). As a consequence, the proportion of patients still treated at 360 days was higher among patients receiving at discharge prasugrel (45/56; 80%) and ticagrelor (117/147; 80%) compared with those receiving clopidogrel (54/92; 59%). No statistically significant difference was observed between patients receiving prasugrel or ticagrelor.

At 360 days, 119/155 (77%) patients under 65 years were still on OAP treatment, compared with 97/140 (69%) patients older than 65 years. The percentage of men (166/224; 74%) and women (50/71; 70%) still treated at 360 days was similar (*Table *3).

**Table 3:** Treatment persistence at 360 days among the persistence population (*n* = 295)

	Count at treatment start	Count at 360 days	Persistence at 360 days (%)
*Age*			
Less than 65 years	155	119	77
More or equal to 65 years	140	97	69
*Gender*			
Male	224	166	74
Female	71	50	70
*ACS type*			
Unstable angina	28	21	75
STEMI	149	117	79
NSTEMI	118	78	66
*ACS management*			
PCI	267	202	76
CABG	6	3	50
PCI + CAGB	4	2	50
Angiography without coronary intervention	7	5	71
Non-invasively managed	11	4	36
*OAP treatment at discharge*			
Clopidogrel	92	54	59
Prasugrel	56	45	80
Ticagrelor	147	117	80
*CV history*			
Prior CAD	178	126	71
Cardiac heart failure	12	5	42
Atrial fibrillation	17	13	76
Stroke/transient ischaemic attack	16	11	69
Peripheral vascular disease	21	14	67
Major bleeding events	2	1	50
*CV risk factors*			
Arterial hypertension or antihypertensive drugs	186	131	70
Dyslipidaemia or cholesterol lowering drugs	209	160	77
Diabetes or hypoglycaemic therapy	70	57	81
Active smoker	112	82	73
Familial history of coronary artery disease	90	68	76
Obesity (BMI >30)	53	40	75
Chronic kidney disease (GFR <60 mg/dl/min)	24	19	79
*Concomitant medication*			
Acetylsalicylic acid	285	210	74
Vitamin K antagonist	10	6	60
LWMH	7	5	71
Dabigatran	1	1	100
Rivaroxaban	4	2	50
None	5	3	60

*n*, number of patients; ACS, acute coronary syndrome; STEMI, ST-segment elevation myocardial infarction; NSTEMI, non-ST-segment elevation myocardial infarction; PCI, percutaneous coronary intervention; CABG, coronary artery by-pass grafting; OAP, oral antiplatelet; CV, cardiovascular; CAD, coronary artery disease; BMI, body mass index; GFR, glomerular filtration rate; LWMH, low molecular weight heparin.

At 360 days, 21/28 (75%) patients diagnosed with UA, 117/149 (79%) patients diagnosed with STEMI and 78/118 (66%) patients diagnosed with NSTEMI were still taking the OAP treatment prescribed at discharge.

Most of the patients managed with PCI (202/267; 76%) and angiography without coronary intervention (5/7; 71%) vs. only half of those managed with coronary artery by-pass grafting (CABG) (3/6; 50%) or PCI and CABG (2/4; 50%) were still on OAP treatment at 360 days. Less than half of the non-invasively managed patients (4/11; 36%) continued OAP treatment at 360 days after discharge from the hospital (*Table *3).

Of the 285 patients taking acetylsalicylic acid at discharge from hospital, 210 (74%) were still using the prescribed OAP treatment at the end of the follow-up period (*Table *3). The majority of patients in the persistence population receiving anticoagulants at discharge (64%; 14/22) continued OAP treatment after 360 days.

### Changes in OAP treatment: reasons and deciders

**Figure 3:**
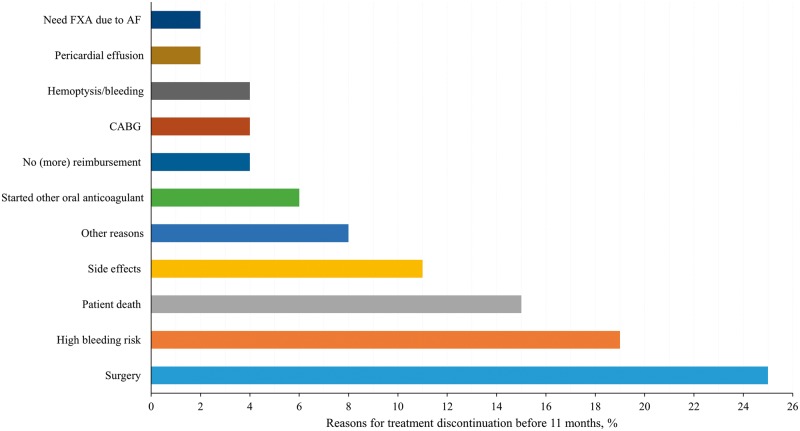
Reasons for stopping OAP treatment before 11 months. OAP, oral antiplatelet; CAGB, coronary artery bypass graft; FXA, coagulation factor X; AF, atrial fibrillation. Note: Other reasons were: standard practice; palliative care; no objective reasons/reasons related to patient.

In the 11-month interval from discharge, 217/295 (74%) of the patients did not change the treatment. Sixty-one out of 295 (21%) patients stopped any OAP treatment before 11 months, 8 (3%) patients stopped and subsequently reinitiated and continued treatment with the same OAP, and 9 (3%) patients continued the treatment with another OAP. The main specific reasons for stopping the treatment were minor surgery (25%) and perceived high-bleeding risk (19%) (*Figure *3). Lack of treatment reimbursement was reported as a reason for OAP discontinuation in only 4% of cases, and 2 (4%) of patients discontinued the treatment of their own accord; however, the reasons for this choice were not recorded in the case report forms. In total, patient death accounted for 15% of treatment cessation, treatment disruption related to the patient (due to non-compliance or bleeding) was recorded in 10% of cases, planned treatment interruption due to an upcoming invasive procedure occurred for 28% of instances, and in 47% of cases the discontinuation was recommended by the physician.

Twelve instances in which the OAP was changed before 11 months were recorded (in 3 instances the treatment was then stopped); 8 (67%) of these treatment switches were triggered by side-effects (non-bleeding complications). Other incidental reasons for OAP switch included cost of treatment (1/12; 8%), high-bleeding risk (1/12; 8%), a new CV event or revascularization (1/12; 8%), or unknown reason (1/12; 8%).

Fifteen OAP treatment re-initiations before 11 months were observed (treatment was stopped again prematurely in 4 instances). About half of the re-initiations occurred after a new CV event (7/15; 47%) and 5 (33%) following surgery. Two patients were re-challenged after side-effects and 2/15 for other reasons.

The decision taker for either treatment stopping or switching to another OAP were in most instances an interventional cardiologist (19/61; 31% and 5/12; 42%, respectively) followed by a non-interventional cardiologist (18/61; 30% and 4/12; 33%, respectively), whereas the decision to re-initiate was in almost half of the instances made by a surgeon (5/11; 45%).

### Multivariate analysis

In a multivariate analysis, ACS management, choice of OAP at discharge, CV history and CV risk were significantly associated with OAP treatment persistence (*Table *4). The type of ACS only showed a trend of association (*P* = 0.0714). Patients treated by PCI were more likely to persist on DAPT than those non-invasively managed (odds ratio = 8.084; *P* = 0.00756). Conversely, patients discharged on clopidogrel were less likely to be adherent to the treatment up to 1 year, when compared with those using ticagrelor (calculated odds ratio = 0.250, *P* = 0.00046).

**Table 4:** Summary of logistical regression analysis for variables predicting OAP treatment persistence with age as continuous variable

Variables	Effects	OR	95% CI	*P*-value
*Age (years)*	–	0.975	0.944–1.006	0.11803
*Gender*	Male vs. female	0.811	0.372–1.770	0.59904
*Hospital type*	Academic vs. non-academic PCI	2.019	0.872–4.674	0.10095
	Non-academic non-PCI vs. non-academic PCI	1.816	0.751–4.391	0.18521
*ACS diagnosis*	Unstable angina vs. NSTEMI	3.728	0.914–15.205	0.06660
	STEMI vs. NSTEMI	1.996	0.942–4.233	0.07140
*ACS management*	PCI vs. non-invasively managed	8.084	1.744–37.471	0.00756
	CABG vs. non-invasively managed	4.747	0.413–54.574	0.21130
	PCI+CABG vs. non-invasively managed	0.862	0.044–17.008	0.92204
	Angiography without coronary intervention vs. non-invasively managed	8.949	0.651–123.099	0.10132
*OAP at discharge*	Clopidogrel vs. ticagrelor	0.250	0.115–0.543	0.00046
	Prasugrel vs. ticagrelor	0.916	0.322–2.608	0.86930
*CV history*^a^	Prior CABG	7.317	1.208–44.314	0.03033
	Congestive heart failure	0.136	0.023–0.790	0.02624
*CV risk factors*^b^	Arterial hypertension or antihypertensive drugs	0.409	0.192–0.869	0.02019
	Dyslipidemia or cholesterol lowering drugs	2.416	1.129–5.174	0.02313
	Diabetes or hypoglycaemic therapy	4.185	1.653–10.595	0.00252

Bolded values indicate significant associations with OAP treatment persistence (*P* < 0.05).

OR, odds ratio; CI, confidence interval; PCI, percutaneous coronary intervention; ACS, acute coronary syndrome; NSTEMI, non-ST-segment elevation myocardial infarction; STEMI, ST-segment elevation myocardial infarction; CABG, coronary artery by-pass grafting; OAP, oral antiplatelet; CV, cardiovascular; MI, myocardial infarction; PCI, percutaneous coronary intervention..

a CV history included 9 categories: prior MI, prior PCI, prior CABG, coronary artery disease and stenosis >50%, congestive heart failure, atrial fibrillation, stroke/transient ischemic attack, peripheral vascular disease and major bleeding events. Only those with significant association are presented.

b CV risk factors included 7 categories: arterial hypertension or antihypertensive drugs, dyslipidaemia or cholesterol lowering drugs, diabetes or anti-diabetes drugs, active smoker, familial history of coronary artery disease, obesity (body mass index >30) and chronic kidney disease. Only those with significant association are presented.

NSTEMI, clopidogrel treatment, congestive heart failure, arterial hypertension, or antihypertensive drugs were all associated with shorter OAP treatment persistence while prior CABG, dyslipidaemia, or cholesterol lowering drugs, diabetes or anti-diabetes drugs were associated with longer OAP treatment persistence (*Table *4).

## Discussion

The main finding of this retrospective study is that the majority of Belgian ACS patients remain adherent to the recommended course of OAP treatment, in line with the European Society of Cardiology guidelines.^2^ Premature OAP treatment discontinuation was observed mainly in the last 3 months of the year following the index event. The discontinuation was more prevalent in patients treated with clopidogrel and in most cases, it was decided by the cardiologist. The most frequent reasons for premature treatment cessation were minor surgery and high bleeding risk.

A systematic review^6^ assessing adherence to DAPT after coronary stenting emphasized an increasingly persistent DAPT use at 12 months starting with the year 2007, when ACC/AHA guidelines were changed to recommend a 12-month duration of DAPT. The use of OAPs at 12 months after drug-eluting stent implementation increased from 63.8% for studies ending between 2004 and 2006 to 78.1% for those ending between 2007 and 2009. A decline in OAP adherence was evidenced by Month 6 of the treatment period.^6^ These trends mirror those of our study: the overall assessed persistence to OAP treatment showed a decrease in time, with more than 90% of the patients still using OAPs at Month 3, to 73% by the end of 1 year of treatment. The lowest persistence at the end of the follow-up period was found for patients receiving clopidogrel (54%), probably due to large extent to their comorbidities, including the more frequent need for oral anticoagulation. The difference between clopidogrel and prasugrel/ticagrelor treatment persistence was not fully explained by data available from the current study, but further investigation might prove worthwhile. The percentages observed at every 90-day interval are similar to those found in a previous study on DAPT medication adherence for clopidogrel and aspirin.^7^ Better medication persistence was found in our study for both prasugrel and ticagrelor at each time point, accompanied by fewer discontinuations between assessments. Similarly to our observations, a recent study in the USA found no difference between adherences to prasugrel and ticagrelor in patients with ACS treated with PCI, while mean persistence was marginally longer with prasugrel than with ticagrelor.^8^

OAP adherence may be influenced by the variety of treatment options, comorbidities, and prior use of the medication, as well as reimbursement restrictions. A study on clopidogrel use after discharge following PCI in the US shows that premature discontinuation was more likely in patients younger than 55 years, in patients having been hospitalized previously or undergoing PCI without stenting, or in patients with chronic obstructive pulmonary diseases and diabetes.^9^ Prior clopidogrel use was also significantly associated with lower adherence, while prior use of β-blockers and statins was associated with longer medication adherence.^9^ In a study assessing adherence to prasugrel treatment following an ACS event in US patients, prior PCI, depression and bleeding were found to be indicative of lower adherence, while baseline statin use predicted better adherence to treatment.^10^ In our study, prior PCI did not significantly impact adherence to treatment, but prior CABG, dyslipidaemia, and diabetes or associated treatment were identified as predictors for OAP treatment persistence, while arterial hypertension or use of antihypertensive drugs were more likely to lead to lower persistence. The type of invasive management was also found to be of importance, as patients with PCI are more likely to display higher medication persistence than those for which the ACS was managed non-invasively. It is likely that a medical, non-invasive approach might lead to patients (or physicians) not realizing the importance of the ACS event. Similar to a previous study on medication persistence among NSTEMI patients,^11^ no significant association was identified between the hospital type (academic vs. non-academic) and medication persistence, regardless of the type of OAP. Although patients treated at academic sites were believed to be more likely to receive medication treatment,^12^ there is no conclusive study showing an improvement in medical adherence.

DAPT discontinuation after PCI was analysed in the PARIS observational study in clinical hospitals in Europe and USA.^13^ The study determined that the main reason for treatment cessation is physician guidance, while bleeding and patient non-compliance was recorded for only 10% of the patients. In line with these observations, in our study, patient-related treatment disruption was identified in 10% of cases and physician-recommended discontinuation accounted for 47% of cases. Physician discretion was also recently shown to account for approximately one-half of reasons for premature cessation of treatment with P2Y_12_ receptor inhibitors following MI, while only in ∼8% of instances a switch to another drug class was recommended.^14^ Our study also identified the cardiologist (either interventional or non-interventional) as the main decision taker for treatment discontinuation, as opposed to the GP or the patient.

The majority of reasons for OAP treatment cessation in this study were objective. Surgery and perceived high-bleeding risk were frequently cited as the driving factor for a shorter DAPT course, in line with contemporary guidelines.^2^ Cost-related reasons for treatment discontinuation following an ACS event have been frequently reported before.^14^^,^^15^ Due to the particularities of the Belgian healthcare system (i.e. compulsory state-based health insurance and universal reimbursement for post-ACS DAPT), cost was found to be of minor importance here, with only 4% of the patients reporting discontinuation of the treatment for lack of reimbursement.

The patients included in the current study had comparable baseline characteristics to other Western European patients included in the EURHOBOP study.^16^ Therefore, the study results can be extended to the Belgian and European population, within the limitations of observational analysis.

The results of this study will hopefully improve the awareness of Belgian physicians and patients on the actual, real-world OAP treatment persistence. Although already relatively high, OAP persistence might still be improved, as premature DAPT treatment discontinuation could be associated with significant risk. Providing the patient with a list of instructions, explaining the reasons and side-effect for each medication and a consistent follow-up planned before discharge have been associated with higher medication persistence after an MI event.^14^

The study has some potential limitations. First, the number of patients with complete dataset included in the analysis was lower compared with the estimated number of patients. Moreover, the patients with complete data are most likely to be those with better adherence to medication and follow-up plan, either induced by the physician or self-motivated, while patients not willing to adhere to the follow-up plan are conceivably less likely to continue treatment as well. The retrospective design of the study was probably the reason for the relatively high rate of missing data,^17^ but at the same time it allowed the collection of accurate “real-life” data on OAP treatment and/or its duration. On the contrary, a prospective follow-up registry has the disadvantage of a positive bias towards a better treatment persistence as both physician and patients are more engaged in the project, and will be probably more aware about the benefit of treatment persistence.

Taken together, a high OAP treatment persistence was observed in Belgian patients throughout the year following an ACS. OAP treatment was prematurely discontinued mainly in the last 3 months of the year following the index event. Discontinuation was observed especially in patients treated with clopidogrel and was most often initiated by the patient’s cardiologist.
